# New feel for new phyla

**DOI:** 10.1111/j.1462-2920.2008.01699.x

**Published:** 2008-08

**Authors:** Michael Y Galperin

**Affiliations:** National Center for Biotechnology Information, National Library of Medicine, National Institutes of HealthBethesda, MD 20894, USA.

According to the dictionary, the Latin term ‘phylum’ comes from Greek *phylon* (ϕũλov), which means ‘race, tribe or clan’ and is unrelated to either *philia* (ϕıλíα) meaning ‘love, affection’ or to the ‘feel’, which comes from Old English *felan* ‘to touch’. These similarly sounding words illustrate a key problem of systematic microbiology: How can we extract useful information from short sequence fragments and not be swayed by superficial similarities? One of the most useful approaches has been binning together sequences from related microorganisms, even if the nature of these organisms remained unknown. This resulted in a number of candidate microbial phyla that still have no cultivated representatives (Hugenholtz *et al*., 1998). Extensive sequencing has been the only way to get a ‘feel’ of these organisms, find out at least some information about their physiology and distribution in the environment. The ultimate goal, of course, is to get a complete genome sequence of the previously uncharacterized organism and use the power of comparative genome analysis to deduce its features.

The past 2 months have been marked by the release of complete genome sequences from first representatives of two new phyla, *Verrucomicrobia* and Candidate division Termite group 1 (Table 1). The first one is now represented by three different genomes, the second one – by two.

**Table 1:** Recently completed microbial genomes (April–May 2008).

Species name	Taxonomy	GenBank accession	Genome size (bp)	Proteins (total)	Sequencing centre	Reference
New organisms						
*Akkermansia muciniphila*	*Verrucomicrobia*	CP001071	2 664 102	2138	JGI	Unpublished
*Methylacidiphilum infernorum*	*Verrucomicrobia*	CP000972	2 287 145	2473	U. Hawaii	Hou *et al*. (2008)
*Opitutus terrae*	*Verrucomicrobia*	CP001032	5 957 605	4612	JGI	Unpublished
Uncultured Termite group 1 bacterium Rs-D17	Candidate division TG1	AP009510-AP009513	1 148 570(total)	776	RIKEN	Hongoh *et al*. (2008)
*Elusimicrobium minutum*	Candidate division TG1	CP001055	1 643 562	1529	JGI	Unpublished
*Corynebacterium urealyticum*	*Actinobacteria*	AM942444	2 369 219	2024	Bielefeld U.	Tauch *et al*. (2008)
*Mycobacterium marinum*	*Actinobacteria*	CP000854	6 636 827	5452	Sanger Institute	Stinear *et al*. (2008)
		CP000895	23 317			
*Streptomyces griseus*	*Actinobacteria*	AP009493	8 545 929	7136	Kitasato U.	Ohnishi *et al*. (2008)
*Nostoc punctiforme*	*Cyanobacteria*	CP001037-CP001042	9 059 191(total)	6690	JGI	Unpublished
*Candidatus* Phytoplasma australiense	*Firmicutes*	AM422018	879 959	684	MPIMG	Tran-Nguyen *et al*. (2008)
*Exiguobacterium sibiricum*	*Firmicutes*	CP001022	3 034 136	3015	JGI	Unpublished
		CP001023	4 885			
		CP001024	1 765			
*Lactobacillus fermentum*	*Firmicutes*	AP008937	2 098 685	1843	Kitasato U.	Morita *et al*. (2008)
*Beijerinckia indica*	*α-Proteobacteria*	CP001016	4 170 153	3784	JGI	Unpublished
		CP001017	181 736			
		CP001018	66 727			
*Burkholderia phymatum*	*β-Proteobacteria*	CP001043-CP001046	8 676 562(total)	7496	JGI	Unpublished
*Burkholderia phytofirmans*	*β-Proteobacteria*	CP001052	4 467 537	7241	JGI	Unpublished
		CP001053	3 625 999			
		CP001054	121 122			
*Stenotrophomonas maltophilia*	*γ-Proteobacteria*	AM743169	4 851 126	4430	Sanger Institute	Crossman *et al*. (2008)
*Borrelia hermsii*	*Spirochaetes*	CP000048	922 307	819	RML-NIAID	Unpublished
*Borrelia turicatae*	*Spirochaetes*	CP000049	917 330	820	RML-NIAID	Unpublished
New strains						
*Porphyromonas gingivalis* ATCC 33277	*Bacteroidetes*	AP009380	2 354 886	2090	Kitasato U.	Naito *et al*. (2008)
*Clostridium botulinum* B str. Eklund 17B	*Firmicutes*	CP001056	3 800 327	3527	JGI	Unpublished
		CP001057	47 642			
*Clostridium botulinum* E3 str. Alaska E43	*Firmicutes*	CP001078	3 659 644		JGI	Unpublished
*Lactobacillus reuteri*F275JCM1112	*Firmicutes*	AP007281	2 039 414	1820	Kitasato U.	Morita *et al*. (2008)
*Streptococcus pneumoniae*CGSP14	*Firmicutes*	CP001033	2 209 198	2206	BGI	Unpublished
*Brucella abortus*S19	*α-Proteobacteria*	CP000887	2 122 487	3000	VBI	Crasta *et al*. (2008)
		CP000888	1 161 449			
*Orientia tsutsugamushi* str. Ikeda	*α-Proteobacteria*	CP000887	2 008 987	1967	Kitasato U.	Nakayama *et al*. (2008)
		CP000888				
*Burkholderia ambifaria*MC40-6	*β-Proteobacteria*	CP001025	7 642 536	6697	JGI	Unpublished
*Acinetobacter baumannii* ACICU	*γ-Proteobacteria*	CP000863	3 904 116	3759	CNR-ISS	Iacono *et al*. (2008)
		CP000865	64 366			
		CP000864	28 279			
*Francisella tularensis* ssp. *mediasiatica*FSC147	*γ-Proteobacteria*	CP000915	1 893 886	1406	JGI	Unpublished
*Shigella boydii* CDC 3083–94	*γ-Proteobacteria*	CP001058-CP001063	4.86(total)	4557	JCVI	Unpublished
*Xanthomonas oryzae* pv. *oryzae* PXO99A	*γ-Proteobacteria*	CP000967	5 240 075	4988	JCVI	Salzberg *et al*. (2008)
*Xylella fastidiosa*M23	*γ-Proteobacteria*	CP001011	2 535 690	2201	JGI	Unpublished
		CP001012	38 297			
*Yersinia pseudotuberculosis*PB1/+	*γ-Proteobacteria*	CP001048	4 695 619	4237	JGI	Unpublished
*Helicobacter pylori* Shi470	*ε-Proteobacteria*	CP001072	1 608 547	1567	Wash U.	Unpublished

Sequencing centre names are abbreviated as follows: BGI, Bejing Genomics Institute, Beijing, China; Bielefeld U., Centrum für Biotechnologie, Universität Bielefeld, Bielefeld, Germany; CNR-ISS, Institute for Biomedical Technologies, National Research Council, Milan, and Istituto Superiore di Sanità, Rome, Italy; JCVI, J. Craig Venter Institute (formerly TIGR), Rockville, Maryland, USA; JGI, US Department of Energy Joint Genome Institute, Walnut Creek, California, USA; Kitasato U. Kitasato Institute for Life Science, Kitasato University, Tokyo, Japan; MPIMG, Max Planck Institute for Molecular Genetics, Berlin, Germany; RIKEN, Genomic Sciences Center, RIKEN, Kanagawa, Japan; RML-NIAID, Rocky Mountain Laboratories, National Institutes of Allergy and Infectious Disease, National Institutes of Health, Hamilton, Montana, USA; Sanger Institute, The Wellcome Trust Sanger Institute, Wellcome Trust Genome Campus, Hinxton, Cambridgeshire, UK; U. Hawaii, Advanced Studies in Genomics, Proteomics and Bioinformatics, University of Hawai'i at Manoa, Honolulu, Hawaii, USA; VBI, Virginia Bioinformatics Institute at Virginia Tech, Blacksburg, Virginia, USA; Wash U., Washington University Medical School, St. Louis, Missouri, USA.

The phylum *Verrucomicrobia,* first recognized as a separate bacterial lineage more than 20 years ago (Albrecht *et al*., 1987; Hedlund *et al*., 1997), remains poorly characterized. Environmental sampling revealed representatives of this phylum in a wide range of environments, including soils, seawater, hot springs and human gastrointestinal tract (Wagner and Horn, 2006). However, few members of *Verrucomicrobia* have been isolated in pure culture and, until recently, there were few sequences from this phylum. To address this deficiency, JGI scientists have launched genome sequencing of five members of *Verrucomicrobia* (see http://www.jgi.doe.gov/sequencing/why/CSP2006/Verrucomicrobia.html). Genomes of two organisms (*Akkermansia muciniphila* and *Opitutus terrae*) have now been completed and three more genomes released in the draft form (Bacterium Ellin514, 7.5 Mbp, GenBank accession number ABOX00000000, *Opitutaceae* bacterium TAV2, 4.9 Mbp, ABEA00000000; and *Verrucomicrobium spinosum*, 8.2 Mbp, ABIZ00000000). A genome of one more member of *Verrucomicrobia*, an extremely acidophilic methanotroph *Methylacidiphilum infernorum*, has been sequenced at the University of Hawaii (Hou *et al*., 2008).

*Akkermansia muciniphila* is a strictly anaerobic bacterium, originally isolated from a human fecal sample, that can use gastric mucin as carbon, energy and nitrogen source (Derrien *et al*., 2004). It has been named after Dutch microbiologist Antoon D.L. Akkermans, professor at Wageningen University and a pioneer in studying molecular ecology of bacterial communities (see http://www.mib.wur.nl/UK/AF/). Recent studies showed that *A. muciniphila* is a common inhabitant of the human intestinal tract, comprising up to 1% of the total bacteria in the intestine (Derrien *et al*., 2008). It grows optimally at 37°C and is capable of fermenting glucose, *N*-acetylglucosamine and *N*-acetylgalactosamine. The genome size of *A. muciniphila* is far smaller than those of other verrucomicrobia (see above), suggesting a massive gene loss in the course of adaptation to the life in nutrient-rich human intestine.

Another sequenced member of *Verrucomicrobia*, *O. terrae*, is also a strictly anaerobic saccharolytic bacterium. It was isolated from a rice paddy soil microcosm, obtained from rice fields in Vercelli, Italy (Chin *et al*., 2001). *Opitutus terrae* can metabolize various mono-, di- and polysaccharides, fermenting them into acetate and propionate.

The third verrucomicrobial genome represents one of the recently characterized methanotrophic strains, mentioned in this column 4 months ago. Three different groups reported independent isolation of extremely acidophilic methanotrophs belonging to the phylum *Verrucomicrobia* from a methane-emitting geothermal field in New Zealand, a Solfatara volcano mudpot in Italy, and from an acidic hot spring in Kamchatka, Russia (Dunfield *et al*., 2007; Pol *et al*., 2007; Islam *et al*., 2008). These three isolates were all thermophiles capable of growing aerobically at 55–60°C with methane as the sole carbon source. They had 98% identical rRNA sequences, indicating that they belong to the same genus, for which the name ‘*Methylacidiphilum’* is being proposed. The complete genome sequence of the New Zealand isolate has now been published (Hou *et al*., 2008). *Methylacidiphilum infernorum* is an autotrophic bacterium whose 2.3 Mbp genome is even smaller than that of *A. muciniphila*. Signs of genome streamlining during adaptation to its unique ecological niche are seen in the organization of central metabolism of *M. infernorum*, including its C1-utilization pathways, simple signal transduction machinery and a limited set of transcriptional regulators (Hou *et al*., 2008).

Verrucomicrobial genomes are very interesting from the evolutionary standpoint. Phylogenetic analysis of *M. infernorum* proteins confirmed earlier conclusions on the proximity of *Verrucomicrobia* and *Chlamydiae* (Hugenholtz *et al*., 1998; Griffiths and Gupta, 2007), which are often treated as a single *Chlamydiae*/*Verrucomicrobia* group. It also provided support for specific association of *Chlamydiae*/*Verrucomicrobia* with *Planctomycetes* and *Lentisphaerae*, and two candidate phyla; *Poribacteria* and OP3, referred to as *Planctomycetes/Verrucomicrobia/Chlamydiae* superphylum (Wagner and Horn, 2006). However, genome analysis did not support the idea of a potential evolutionary relationship between *Verrucomicrobia* and eukaryotes, which had been prompted by the discovery of tubulin in members of the genus *Prosthecobacter*, also belonging to the *Verrucomicrobia* (Jenkins *et al*., 2002; Staley *et al*., 2005). The genome of *M. infernorum* did not encode tubulin or, for that matter, close homologues of any other signature eukaryotic proteins (Hou *et al*., 2008)*.* Tubulin genes were missing also in *A. muciniphila* and *O. terrae* genomes. These results argue against bacterial origin of tubulin and suggest that *Prosthecobacter* acquired tubulin genes through lateral gene transfer from some eukaryotic cells after its divergence from other verrucomicrobial lineages.

The second new phylum with recently sequenced genomes, candidate division ‘Termite group I’ (TG-1), includes no cultivated representatives (however, see below) and has been defined on the basis of rRNA sequences obtain by environmental sampling (Hugenholtz *et al*., 1998). Representatives of one TG-1 lineage, the so-called *“Endomicrobia”*, are abundant in the termite gut, where they are found as intracellular symbionts of various wood-feeding protozoa (Stingl *et al*., 2005; Ikeda-Ohtsubo *et al*., 2007). TG-1 representatives can also be detected in many other habitats, including rice soil, river sediment and cow rumen (Herlemann *et al*., 2007b; Ohkuma *et al*., 2007). Although there have been no physiological studies of any TG-1 member, the conditions inside the termite gut suggest that they are obligately anaerobic bacteria that gain energy by fermentation of wood-derived carbohydrates.

Now, after many years of having just bits and pieces of TG-1 sequences, we suddenly have two completely sequenced genomes of TG-1 members. The first of them comes from bacterial phylotype Rs-D17, a member of the *“Endomicrobia”*, which is found specifically within the cells of the cellulolytic flagellate *Trichonympha agilis* that inhabits the gut of the termite *Reticulitermes speratus* (Hongoh *et al*., 2008). By using as the DNA source only ∼10^3^ bacterial cells isolated from a single cell of *T. agilis*, it became possible to obtain sufficiently pure and uniform population to perform the sequencing and assembly of Rs-D17 genome. The reconstructed genome consists of a circular 1.1 Mbp chromosome and three plasmids of 11.6, 5.7 and 5.3 kb. It shows clear signs of genome streamlining, including presence of numerous pseudogenes and partial or complete loss of certain metabolic pathways. Still, cells of Rs-D17 appear to be able to synthesize at least 15 amino acids, purines and pyrimidines (Hongoh *et al*., 2008). The authors suggest that Rs-D17 serves as an intracellular symbiont of *T. agilis*, supplying the host protist cell with amino acids and vitamins more or less the same way as it happens in *Buchnera*-aphid symbiosis.

The second TG-1-related genome comes from *Elusimicrobium minutum* Pei191, the first cultivated representative of this phylum, which still remains to be formally described. According to Andreas Brune and colleagues at Max Planck Institute for Terrestrial Microbiology in Marburg, Germany, *E. minutum* is an obligately anaerobic ultra-microbacterium (0.2–0.3 μm in diameter) that was isolated from sterile-filtered gut homogenates of the larva of humivorous scarab beetle *Pachnoda ephippiata* (*Coleoptera*: *Scarabaeidae*; see Egert *et al*., 2003; Lemke *et al*., 2003). This organism grows heterotrophically on glucose and produces acetate, hydrogen and ethanol as major products (Herlemann *et al*., 2007a). It belongs to the so-called ‘Intestinal Cluster’, which represents a distinct lineage of TG-1-affiliated microorganisms present in arthropod guts and in the cow rumen (Herlemann *et al*., 2007b). The relatively small genome size of both *E. minutum* and Rs-D17 may reflect their adaptation to gut environment and is not necessarily representative of the whole TG-1 group.

There have also been interesting genomes among relatively well-known bacterial phyla. *Actinobacteria*, for example, are represented by three new genomes coming from opportunistic human pathogens *Corynebacterium urealyticum* and *Mycobacterium marinum* and the soil bacterium *Streptomyces griseus*, the original producer of streptomycin.

Although *C. urealyticum* is part of the natural flora of human skin, it often colonizes the urinary tract, causing a variety of urinary tract infections. Presence of *C. urealyticum* in urine samples correlates with elevated pH and presence of struvite (magnesium ammonium phosphate hexahydrate) stones. The sequenced strain *C. urealyticum* DSM7109 was originally isolated from a patient with alkaline-encrusted cystitis. Its growth requires presence of exogenous lipids, explained by the absence of a fatty acid synthase gene and presence of a robust system for degradation of exogenous fatty acids (Tauch *et al*., 2008). Presence of several antibiotic-resistance determinants suggests high incidents of lateral gene transfers, which lead to the accumulation of multidrug-resistant strains.

*Mycobacterium marinum* is close relative of *M. tuberculosis* that causes a tuberculosis-like disease in fish and amphibia. Owing to its lower temperature optimum (25–35°C) and a much faster growth than *M. tuberculosis,* it is often used as a convenient experimental model to study tuberculosis in humans (Tobin and Ramakrishnan, 2008). However, *M. marinum* can also infect humans, causing granulomatous skin disease. Comparison of mycobacterial genomes suggests that evolution of *M. tuberculosis* from a *M. marinum*-like ancestral form included reduction in the genome size, accompanied by specialization toward human host and the loss of the ability to survive in the environment (Stinear *et al*., 2008).

The sequenced strain of *S. griseus,* IFO 13350, came from the Waksman laboratory at Rutgers University and is one of the original strains used for production of streptomycin (Ohnishi *et al*., 2008). Analysis of its genome revealed a significant degree of colinearity with genomes of *Streptomyces coelicolor* A3(2) and *Streptomyces avermitilis* with at least 45% of proteins shared by all three genomes. It also identified 34 clusters of genes encoding polyketide synthases and non-ribosomal peptide synthetases. Some of these clusters are responsible for production of known secondary metabolites (streptomycin, grixazone, melanin, carotenoids, siderophores, lantibiotics), products of others remain unknown (Ohnishi *et al*., 2008).

*Nostoc punctiforme* is a nitrogen-fixing terrestrial filamentous cyanobacterium that is closely related to *Anabaena* (*Nostoc*) PCC 7120. This organism can exist in a free state but readily forms symbioses with a wide variety of plants and fungi. It is a favourite model organism for studies of cyanobacterial growth, metabolism, cell development and symbiotic behaviour (Meeks *et al*., 2001; 2002; Meeks, 2006). *Nostoc punctiforme* has one of the most complex developmental programs known in bacteria: in addition to usual vegetative cells, it is capable of producing three kinds of differentiated cells: (i) heterocysts (5–10 μm in diameter) that are surrounded by a thick cell wall and maintain microoxic conditions, allowing fixation of atmospheric nitrogen; (ii) short, motile hormogonium filaments (1.5–2 μm); and (iii) large spore-like akinetes that can reach 15–20 μm in diameter (Meeks *et al*., 2002). The sequenced strain *N. punctiforme* PCC 73102 was isolated from a symbiotic association with the gymnosperm plant *Macrozamia* sp. Its genome consists of an 8.2 Mb chromosome and five plasmids, which range in size from 26 to 354 kb, and encodes an unusually high variety of complex multidomain signaling proteins.

‘*Candidatus* Phytoplasma australiense’ is a mycoplasmal phytopathogen that causes several plant diseases, such as dieback in papaya and Australian grapevine yellows in grapevine (Davis *et al*., 1997). This organism is also remarkable for the place where it is studied, Charles Darwin University in Darwin, Northern Territory, Australia (Tran-Nguyen *et al*., 2008). This university (http://www.cdu.edu.au/) was established in 2003 through merger of several local colleges in Darwin area and is currently the only college in the world named after the great scientist. Politicians, including those that opposed teaching evolution, fared much better.

*Exiguobacterium sibiricum* is a facultatively aerobic non-spore-forming representative of the family *Bacillaceae.* It was isolated from a permafrost core in the Kolyma-Indigirka lowland in Siberia from a depth of 43 m. This depth corresponds to a geological layer estimated to be 2–3 million years old (Rodrigues *et al*., 2006) and unaffected by all the turmoil in that area during the past century. The sequenced strain *E. sibiricum* 255-15 was able to grow at temperatures ranging from −6°C to +40°C and was able to survive long-term freeze and repeated freeze-thawing treatments (Vishnivetskaya *et al*., 2007).

Other interesting organisms with recently sequenced genomes include the nitrogen-fixing acidophilic α-proteobacterium *Beijerinckia indica* ssp. *indica*, a non-methanotrophic bacterium that is closely related to the methanotroph *Methylocella silvestris* (Dunfield *et al*., 2003); plant growth-promoting β-proteobacteria *Burkholderia phymatum* (Elliott *et al*., 2007) and *Burkholderia phytofirmans* (Sessitsch *et al*., 2005); and the soil γ-proteobacterium *Stenotrophomonas maltophilia*, an opportunistic human pathogen that is closely related to the phytopathogenic xanthomonads (Crossman *et al*., 2008).

In other genomics news, it is worth noting two publications that attempt to encourage community involvement in improving the genomic databases.

One of them, produced by the Genomics Standards Consortium (Field *et al*., 2008), introduces the ‘minimum information about a genome sequence’ (MIGS) specification, a common-sense list of parameters that should be reported for each sequenced genome. This list includes, among others, the geographic location and time of the sample collection (plus depth or altitude, if appropriate) and properties of the habitat (temperature, pH, salinity, pressure, light intensity, dissolved organic carbon, dissolved oxygen, phosphate, nitrate, sulfates, sulfides, and so on). While this sounds like a sensible recommendation, this paper does not clearly articulate the penalties, if any, for non-compliance. Besides, what should one do with the isolate for which such data are unavailable, refrain from sequencing the genome or delay the release of the genome sequence until such data become available? Strict adherence to the MIGS standards would have prevented or greatly delayed public release of the genome of *E. minutum,* discussed above, as well as many other genomes sequenced at the JGI and other institutions.

The second paper (Mons *et al*., 2008), whose authors include, among others, Wikipedia founders Jimmy Wales and Erik Moeller, announces creation of a Wiki-based system called WikiProteins, intended for ‘community annotation of biomedical concepts and their interactions’. The core of the system is based on ‘protein concepts’ (in plain language, extended protein annotations) from Swiss-Prot and on Unified Medical Language System (UMLS®) concepts for computer processing of natural language-based biomedical information (see http://www.nlm.nih.gov/pubs/factsheets/umls.html). In the future, WikiProteins are expected to incorporate the Gene Ontology (GO) vocabulary and a variety of other databases. This sounds like a very promising undertaking, and the whole paper (which is freely available online with a variety of colourful links and pop-up windows) deserves a careful reading, even if the idea of ‘collaborative knowledge discovery’ might seem too far-fetched to most readers of this journal.

In conclusion, a correction: in the previous Genomics Update (Galperin, 2008), I confused properties of two methylotrophs. *Methylobacterium* spp. 4-46 is a symbiont of the legume *Lotononis bainesii*, whereas *Methylobacterium radiotolerans* is not known for symbiosis. I thank Benjamin Gourion (ETH Zürich) and Ludmila Chistoserdova (University of Washington) for pointing out this mistake.
